# An Analysis of Respiration with the Smart Sensor SENSIRIB in Patients Undergoing Thoracic Surgery

**DOI:** 10.3390/s22041561

**Published:** 2022-02-17

**Authors:** Marco Ceccarelli, Riccardo Taje, Paula Elena Papuc, Vincenzo Ambrogi

**Affiliations:** 1 LARM2: Lab of Robot Mechatronics, Department of Industrial Engineering, University of Rome Tor Vergata, 00133 Rome, Italy; 2 Department of Surgical Sciences, University of Rome Tor Vergata, 00133 Rome, Italy; riccardo.taje@alumni.uniroma2.eu (R.T.); paulaelena.papuc@alumni.uniroma2.eu (P.E.P.); ambrogi@uniroma2.it (V.A.)

**Keywords:** medical sensors, smart sensors, evaluation of respiration, rib motions, thorax operation patients

## Abstract

The paper examines the problem of respiration monitoring with easily wearable instrumentation by using a smart device that is properly designed and implemented with small and light components. The practical implementation is presented both in practical aspects and from experimental results by following a properly defined method with a medical-like protocol and specific procedure of testing. The results of a statistically significant campaign of experimental tests are reported with the characteristic data from the angles and acceleration components of a sensed rib both to validate the smart device and the procedure for respiration monitoring.

## 1. Introduction

Breathing is a vital act to allow the body to supply itself with air and is characterized by motion acts with biomechanical aspects as well as physiological and chemical functions. The biomechanical aspects refer to the motion that is imposed to the thoracic cavity by the expansion and contraction of the lung tissues and these movements produce kinematic and dynamic aspects that must be coordinated for the correct functionality of the organism. Generally, the biomechanics of the respiratory act are analyzed from a descriptive and not a quantitative point of view, considering only the functionality in general terms from a clinical point of view.

Thoracic surgery significantly affects the pulmonary function. Thoracic wall incisions, iatrogenic pneumothorax with single lung pulmonary ventilation and parenchymal resections radically change the respiratory dynamic. Pulmonary resections are followed by respiratory physiology changes that reflect the loss of pulmonary parenchyma [1,2,3]. Post-operative pain actively participates in these changes, hindering coughing and leading to post-operative persistent atelectasis, pneumonia and eventually lung failure [4,5]. A fixed hemithorax can be the early hallmark of all of these complications. Strategies to evaluate the chest wall motion and respiratory rate are not complete and are generally based on a clinical observation, which may be affected by the experience of the clinician and may require the continuous re-evaluation of a patient [6]. Therefore, automated devices for chest wall motion analyses may help to standardize the early detection of post-operative complications independently from clinician experience. In this context, different devices have been used to evaluate the chest wall motion but none of these devices passed the experimental phase [7]. Other techniques have been developed based on optics [8,9] as well as on acceleration sensing of the thorax motion [10,11]. All these devices are aimed at analyzing respiration in terms of general parameters. Nonetheless, other techniques such as computed tomography dynamic scans or magnetic resonance imaging have been described in the thoracic biomechanical characterization of the thorax [12,13]. Most of these techniques have not been clinically applied due to the expense as well as the invasiveness or encumbrance of the necessary armamentarium. A new, inexpensive non-invasive device based on an inertial measurement unit (IMU) sensor named SENSIRIB has been proposed to assess the respiratory chest wall motion [14,15]. Hereafter, the results and feasibility of this technique are presented to analyze the respiratory changes in the chest wall motion in patients undergoing thoracic surgery before and after surgical procedures. The ability of the device to detect early respiratory disarrangement due to post-operative complications and its reliability in respiratory rate monitoring are described with a quantitative and qualitative evaluation of the respiration acts.

## 2. Materials and Methods

### 2.1. Device Description

The proposed SENSIRIB device is a portable device for measuring the motion characteristics of an individual human rib during respiratory acts. The device structure is designed to be a portable structure using small components with cables for data transmission that can be stored and viewed on a computer or tablet as a display unit.

**Figure 1:**
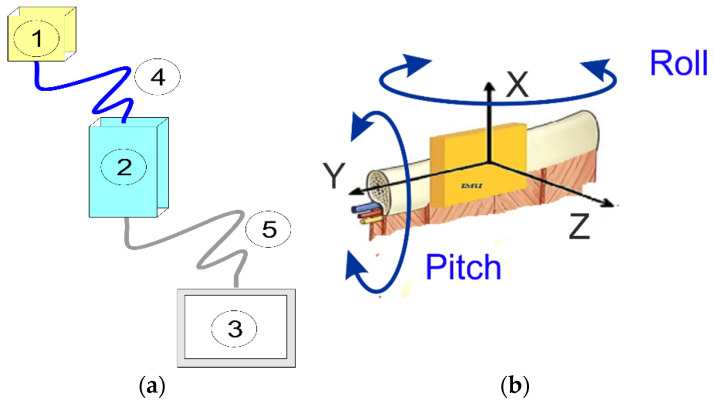
The conceptual design of a SENSIRIB device: (**a**) the scheme; (**b**) the IMU sensor characteristics.

**Figure 2:**
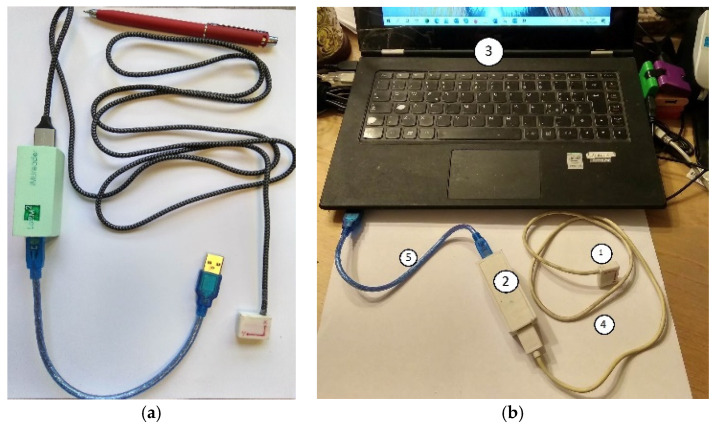
A built prototype of a SENSIRIB device: (**a**) the portable mechanical design; (**b**) the connection to a laptop.

The structure of the SENSIRIB device is described in Figure 1; Figure 1a shows a conceptual scheme of the device with its components and Figure 1b explains the device concept in detecting the rib motion using an IMU (inertial measurement unit). The smart device SENSIRIB is composed of a motion IMU sensor (1), a signal acquisition and processing unit (2), a data display and storage unit (3), a connection cable and signal transmission (4) and a connection cable and data transmission (5) (Figure 1 and Figure 2). Its structure is designed with components that are of a modest size and portable to allow the measurement of movements of a single rib of the human body on which the sensor (1) can be installed. The IMU sensor (1), which is inside a small box, measures the angles and accelerations of the motion of a rib during respiration when it is placed on a human rib with proper skin adhesion. The acquisition and processing of the signals from the IMU sensor in (1) is computed in the signal acquisition and processing unit (2), which has small dimensions so that it can be placed comfortably at a distance on a belt on the human user whose rib movement is under measurement. The connection data transmission cables (4) and (5) are of suitable lengths for positioning units (2) and (3) at a convenient short distance from the user under testing.

Figure 2 shows a built prototype of the smart device at the LARM2 laboratory in Rome, Italy. As can be seen in Figure 2a, the IMU sensor unit (1) is in a small box 1.2 × 1.2 × 0.4 cm with a cable (4) of about 1.2 m connecting to the box of the electronic unit (2). Unit (2) is a box 10.0 × 4.0 × 3.0 cm with a cable (5) to connect to a laptop (3) via a USB port. Figure 2b shows a portable set-up with a laptop for testing activity. The registration and elaboration of the data were performed using LabMonitor Plotter V1.2, which is a software code that has been developed specifically for the SENSIRIB. Minimal invasiveness is ensured by the reduced dimensions of the extremity sensor (1). The portability is ensured by the small components of the structure, which are connected by data transmission cables. The device has a user-oriented operation and is programmed with specific software codes for easy operation from a laptop keyboard with an online display of the acquired data as well as the subsequent processing of the numerical evaluations for a medical diagnosis. The prototype was produced in a small number of units for experimental activity in order to verify the versatility of the device, especially in a respiration evaluation and a user-oriented operation without specific technical skills.

### 2.2. Biomechanics of Respiration

**Figure 3:**
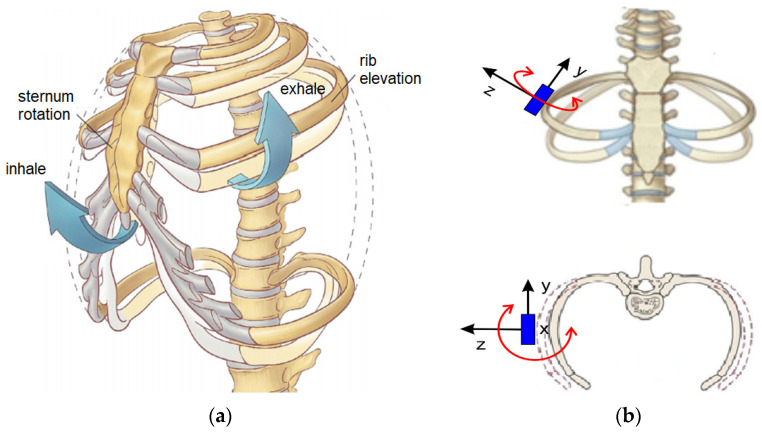
A scheme of the biomechanics of respiration: (**a**) the ribcage thorax motions; (**b**) sensed rib motions.

The biomechanics of respiration can be analyzed by considering the ventilatory act as being related to the movements of the thorax following the expansion of the lung tissues in coordination with the activities of the muscles of the thoracic cavity (Figure 3). The respiratory cycle is a continuous alternation of inspiration and expiration with the motion of the ribcage of the chest; the ribs in particular are subjected to significant motions, as summarized in the sketch in Figure 3b.

The anatomy of the thoracic cavity is characterized by essential elements consisting of the bone structure with cartilage connecting to the bone bodies of the sternum and spine together with the muscle bands. The muscle complex activates the movement of the chest and together with the bone skeleton they also work as a protective function of the organs contained within the chest cavity. Most of the twelve pairs of ribs are connected with their heads posterior to the thoracic vertebrae and anterior to the sternum (throughout the cartilage interposition) to compose the chest wall skeleton (Figure 3a).

The ventilatory act is characterized by kinematic aspects that are related to the movements of expansion and contraction of the rib cage with the corresponding movements of the ribs, as summarized in Figure 3b. The dynamic aspects due to the forces are less evident despite the high constraint reactions that can be produced at the cartilaginous connections of the ribs on the sternum and on the vertebrae. Therefore, a ventilatory act can be mainly characterized by the kinematic aspects of the expansion and contraction motions of the chest ribcage but also by the transmission of those motions to single ribs. Figure 3b summarizes the type of movements that a rib—or more specifically, a rib segment—can generally experience. In particular, the main motions of a rib can be recognized in the elongation movement that is also absorbed by the cartilage tissues at the extremity as well as small twists along the axis of the rib but, above all, the flexion motions that tend to increase the volume of the rib cage with the displacement of the ribs. Referring to these movements, it is also possible to recognize the actions as the mechanical reactions that can be generated in terms of force at the vertebrae to which they are connected. According to medical representations and as shown in Figure 3b, the main movements are represented considering the so-called bucket handle motion combined with a pump handle motion in the top scheme and a caliper motion in the bottom scheme, which are responsible for the variation of the volume of the chest cavity during a respiration act. Thus, a respiration act can be evaluated through the movement of the ribs and the sixth rib can be considered to be the most convenient one for the most significant detectable movements, as in the schemes of Figure 3b.

In summary, the problem of an evaluation of the biomechanics of respiration can be solved with an analysis of the movements of a more mobile rib, which can give significant indications of the state of the ventilatory act during respiration by monitoring the rib movements during the expansion and contraction of the rib cage, as represented in the models of Figure 3.

### 2.3. Measurement Strategy

**Figure 4:**
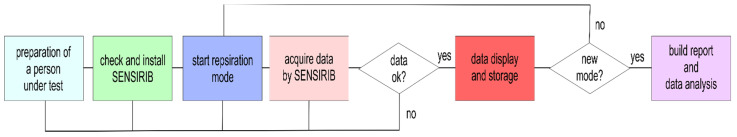
A flowchart for the framework and operation of testing respiration by using SENSIRIB.

Figure 4 summarizes the procedure for a test in a step-by-step procedure according to the following medical protocols: the person under testing is informed of the procedure and is asked for informed agreement to the testing; the IMU sensor is installed on the sixth rib of the person and the cable connections are adjusted for the whole SENSIRIB device linked to a laptop; the SENSIRIB is checked and then started; the person is asked for a respiration mode according to the planned testing; the person activates the respiration mode in the SENSIRIB that acquires the data; the operator checks the data on the laptop display and decides on the valuable results and if there are none, the testing is restarted at one of the previous steps where the operator may have identified the flaws; once the data are considered valuable, they are displayed and stored; these steps are repeated for all the respiration modes planned; finally, a report is built with the acquired data in the form of plots and a data table that will be used for an analysis of the respiration of the person, even from a medical diagnostic viewpoint.

**Figure 5:**
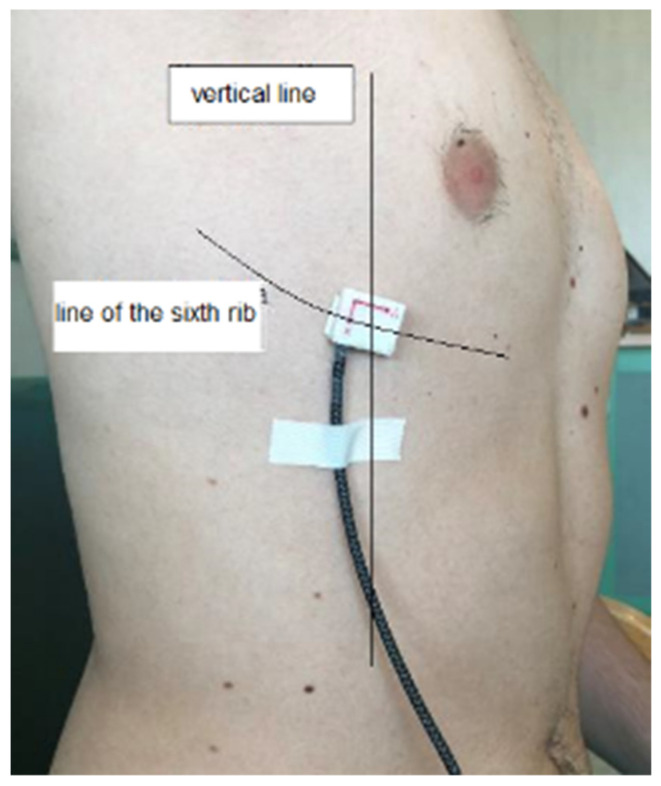
The IMU sensor unit of SENSIRIB on the sixth rib of a volunteer for a test.

A chest wall motion analysis was conducted during basal/maximal respiration and a cough before and after the surgical procedure at day 1. All tests were performed on patients seated with their back resting on a rigid support in order to minimize posterior movements during ventilation. Before each test, the device was calibrated by checking the gravity force value along the axis directions. The IMU sensor was fixed along the right sixth rib at the point crossing the anterior axillary line (Figure 5). This anatomical positioning was chosen because of its centrality in the antero-lateral hemithorax. The sensor was always orientated in the same way. The patients were asked to raise their upper limb to decrease the fat tissue layer and to breathe normally. Considering that the time of each single test was about one minute, once calibrated at the beginning of the test, the IMU sensor could be considered not to be affected by a drift in the data acquisition for the angles and acceleration components of the sensed rib.

The rib motions were recorded during three basal respiratory acts. Each patient was then asked to take three consecutive maximal breaths. Thereafter, the patients were asked to cough three times consecutively. According to this pattern (basal–maximal–cough), three consecutive similar actions were measured with a pause of 15 min. Therefore, each patient underwent measurements of at least three complete respiratory patterns composed of basal/maximal breathing and a cough. The same procedure was repeated after the surgical procedure at day 1. During each test, five biomechanical parameters were recorded and analyzed such as acceleration along the three axes and roll and pitch angles, as seen in the scheme in Figure 1b.

**Figure 6:**
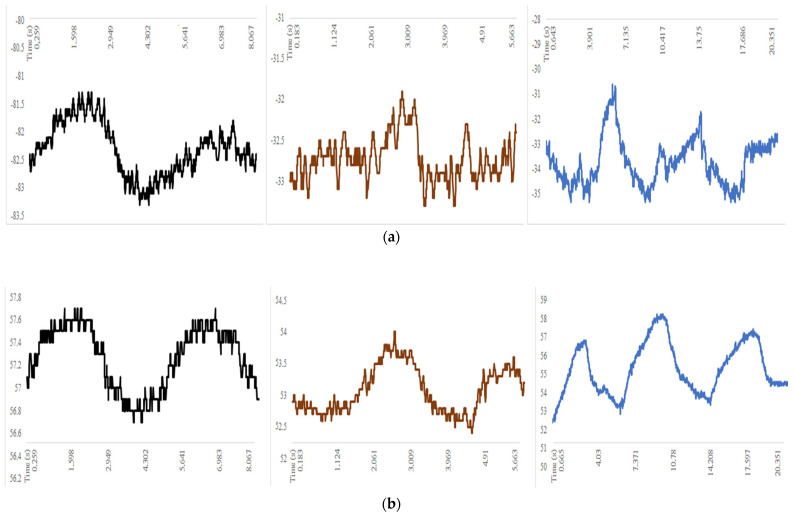
Acquired data by SENSIRIB on the sixth rib on a volunteer for a test in terms of: (**a**) roll angle; (**b**) pitch angle (in black for the pre-operative condition, in red for the post-operative condition on the operated side, in blue for the post-operative condition on the non-operated side).

**Figure 7:**
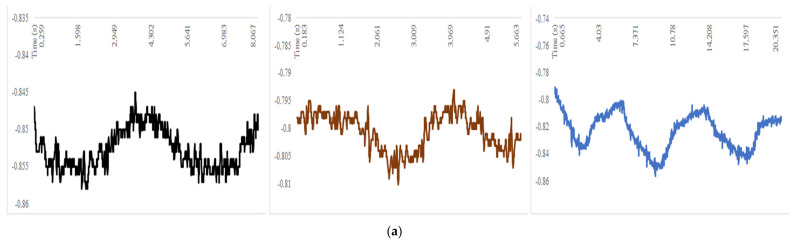

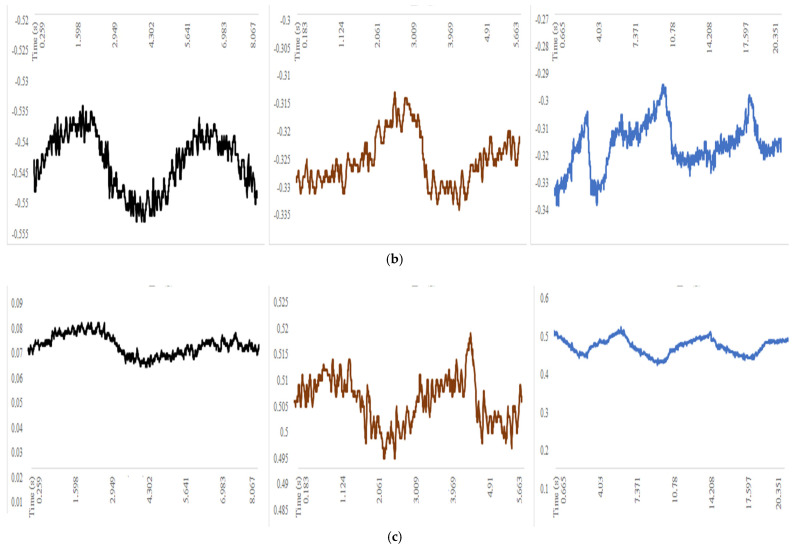
Acquired data by SENSIRIB on the sixth rib on a volunteer for a test in terms of: (**a**) acceleration along X; (**b**) acceleration along Y; (**c**) acceleration along Z (in black for the pre-operative condition, in red for the post-operative condition on the operated side, in blue for the post-operative condition on the non-operated side).

The orientation of the axes in the space was arranged according to the design scheme with the installation set-up of Figure 5. The patient was seated with the sensor positioned on the sixth rib along the anterior axillary line with the reference frame according to Figure 1b and Figure 5. The data recorded in these three different axes helped to three-dimensionally analyze the chest wall motion. Acceleration decomposition into the aforementioned three components (X, Y, Z) and rib angles were separately analyzed; the results are shown in the examples in Figure 6 and Figure 7.

The roll and pitch angles were measured during basal and maximal breathing as well as coughing. An example is shown in Figure 6 where the representative cycles were reported from the full test data referring to the pre-operative condition, post-operative condition on the operated side and post-operative condition on the non-operated side, respectively. The roll angle expressed the angle that a rib reached in the caliper movement around the X-axis during respiration (Figure 3), indicating an oscillating motion around the transversal or cranio-caudal axis of the rib. The pitch angle expressed the angle that a rib reached in the bucket handle movement along the Y-axis during respiration (Figure 3), indicating an oscillating motion around the longitudinal axis of the rib. Referring to Figure 7, the X-component of acceleration was due to a cranio-caudal rib movement during respiration (Figure 3b); the Y-component of acceleration resulted from an antero-posterior rib movement during respiration and the Z-component of acceleration was due to a latero-lateral rib movement during respiration.

### 2.4. Data Storage and Analysis

The data referring to the measured parameters were elaborated on in Excel data sheets. For each parameter, a quantitative analysis was conducted and the average (mean) and standard deviation (SD) were computed, as reported in the illustrative example in Table 1 for one person. From the quantitative data, each parameter was plotted to qualitatively analyze the trend during the time-sequential breaths. The primary objective of the investigation was to enlighten the biomechanical changes before and after a surgical intervention. The secondary objectives were to assess the ability of the SENSIRIB in detecting a post-operative complication and the reliability of the device in estimating respiratory rates.

## 3. Results

### 3.1. Results in the Post-Operative Functional Assessment

Figure 6 and Figure 7 show the results of a characteristic test that was performed before and after a surgical intervention at day 1. Significant changes between the pre-operative and post-operative results were detected, as shown in the reported plots of the illustrative example. A smaller acceleration along the X-axis with a complementary larger acceleration along the Y-axis were detected in most of the patients. In addition, the range of movement in the measured roll and pitch angles was reduced after surgery, as indicated by the standard deviations. A qualitative analysis of the plots in Figure 6 and Figure 7 showed an irregular pattern that characterized the post-operative data. Several picks were noted in both the end-inspiratory and end-expiratory phases. Shortened breaths were associated with a longer plateau and could be identified in the post-operative period. Nonetheless, if the first, second and third respiratory acts were separately compared with the post-operative curve, a high heterogeneity could be found. As shown in Figure 6 and Figure 7, the post-operative respiratory acts were different in both intensity and shape in the data plots. The first respiratory act in particular gave a shape of plot similar to the pre-operative plot even if it was reduced in intensity. Conversely, the second and the third respiratory acts were experienced with prolonged inspiratory phases followed by rapid expiratory phases.

### 3.2. Results in the Complication Prediction

The results of the SENSIRIB application in the early detection of post-operative complications were investigated by comparing the data recorded pre-operatively with the data recorded on the hemithorax on the first post-operative day. As shown in Figure 6 and Figure 7 and Table 1, a biomechanical analysis revealed a post-operative atelectasis of the operated hemithorax. When the values recorded on the operated hemithorax before and after the surgical procedure were compared, a post-operative reduction in both the average values and standard deviation of all the measured parameters was detected. The ranges of the roll angle and pitch angle were both greatly reduced. A qualitative analysis of the data plots showed an irregular pattern in the post-operative curve. The inspiratory and expiratory period could not be clearly recognized in the roll angle plot curve. This chaotic appearance of the plot curve was underlined in all the post-operative curves of patients undergoing post-operative complications. Nonetheless, in the pitch angle plot and the acceleration along the X-axis plot, a reduced respiratory rate as well as reduced end-inspiratory and end-expiratory picks were observable. Conversely, when the data recorded on the non-operated hemithorax in the post-operative period were compared with the pre-operative biomechanical analysis, similar values were detected in the acceleration along the X-axis and in the pitch angle average value for the movement range. Conversely, the roll angle was detected to be reduced in both the average value and movement range in the post-operative period. As predicted by the biomechanical analysis, a post-operative chest X-ray showed a complete atelectasis of the operated lung.

### 3.3. Respiratory Rate Monitoring

**Figure 8:**
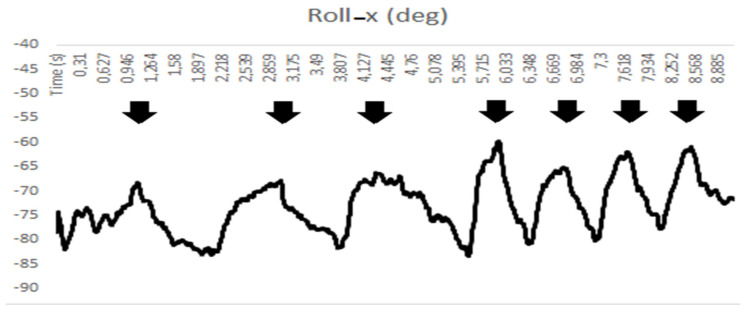
Acquired data by SENSIRIB used for computing the respiratory rate.

The respiratory rate could be evaluated using the data acquired by the SENSIRIB. In the measured time span, the respiratory rate could easily be estimated by counting the expiratory picks of the curve derived from the basal respiration measurements (as shown in Figure 8) and could be recognized in all the measured motion parameters. As a mean of three consecutive acts, the respiratory rate could be computed by:

(1) $$\mathrm{respiratory}\;\mathrm{rate}=\frac{\mathrm{number}\;\mathrm{of}\;\mathrm{respiratory}\;\mathrm{acts}}{\mathrm{time}\;\mathrm{of}\;\mathrm{the}\;\mathrm{final}\;\mathrm{respiratory}\;\mathrm{pick}}.$$

The respiratory rate is recognized a significant measurement in medicine as a fundamental diagnostic tool as indicated in [16,17,18]. Even if different strategies to estimate this parameter have been validated as pointed out in [18], respiratory rate is still neglected, and its clinical evaluation is still based on bed side observation. SENSIRIB can easily and eventually remotely measure respiratory rate and its changing within a biomechanical analysis of respiration.

For the reported example in Figure 6 and Figure 7, and referring to the specific analysis in Figure 8, the respiration rate ranged from 0.3 to 1.1 act/s but with a more variable frequency range.

In a longer acquisition, the variation during normal or tachypneic respiratory acts could be easily assessed and quantified and the respiratory rate could be monitored using the data from the SENSIRIB acquisition (Figure 8).

The SENSIRIB device was used considering the clinical–medical purposes within a protocol not only for standardized use in clear sequential steps but also in respect of the medical–ethical conditions in interactions with human users. The experimental validation was performed with a medical–clinical testing campaign at the thoracic surgery unit in Polyclinic Tor Vergata, Rome, Italy, using thirty volunteer patient subjects after informed consent was obtained according to a predetermined testing protocol.

Figure 6, Figure 7 and Figure 8 and Table 1 show the illustrative results of a characteristic test to demonstrate both the efficiency of the device and a successful characterization of the respiration act that could be performed with the SENSIRIB device.

## 4. Discussion

In the present analysis, a new inexpensive and non-invasive device based on an inertial measurement unit was employed to investigate chest wall movements during respiration in patients undergoing general thoracic surgery procedures. As the first objective of the investigation, chest wall biomechanics were evaluated for changes following pulmonary resections. In addition, the ability of the device was experienced as a non-invasive evaluation of respiratory acts and an example of the respiratory biomechanical pattern was analyzed from the data recorded by the SENSIRIB following a post-operative complication.

### 4.1. Post-Operative Functional Assessment

The comparison of the pre-operative and post-operative data obtained through the SENSIRIB demonstrated a reduction in the acceleration along the X-axis and a compensatory augmentation of the acceleration along the Y-axis in the post-operative period. In addition, a reduction of both the roll and pitch ranges was detected. These results may be explained both as an antalgic response to the surgical procedure and as an effort of the respiratory system to compensate for post-operative diaphragmatic palsy. Pain as well as an inefficient diaphragmatic contraction leads to the activation of the accessory inspiratory muscles in order to maintain an adequate oxygenation. Respiration involves two principal biomechanical patterns, which may be summarized by diaphragmatic-centered respiration and intercostal and accessory muscle-driven respiration. During shallow breathing or in a pre-operative setting, respiration is mainly guided by diaphragm contractions that dislocate downward the abdominal organs and enlarge the pleural cavity, reducing intrapleural pressure and leading to the inspiratory act; diaphragmatic relaxation leads to the expiratory act. In addition, a rib movement is influenced by several anatomical vincula. During diaphragmatic respiration, the rib movement has a double fulcrum between the sternum and column. Thus, the upper chest rib rotates upward and downward in a movement usually defined as a bucket handle movement. Therefore, diaphragmatic respiration influences both the acceleration along the X-axis and, due to the rotational component of the rib movement, determines the pitch angle variation. The X acceleration and pitch angle both decrease after a surgery intervention, confirming the effect of the diaphragmatic contraction in post-operation conditions.

Conversely, intercostal—and particularly, accessory—muscles participate in intense respiration under exertion. Following an accessory muscle contraction, the sternum and anterior component of the chest wall is anteriorly elevated in a movement usually called the pump handle movement. In this situation, the sternum rib has a unique fulcrum at the costo-vertebral joint. The anterior movement of the rib is associated with a rotational component that is laterally directed. As the analysis of the SENSIRIB was centered on the antero-medial portion of the sixth rib, the pump handle movement could be decomposed in an anterior and in a lateral component. The anterior component could be interpretated as the acceleration along the Y-axis whilst the lateral component could be interpreted as the acceleration along the Z-axis. The angle subtending this complex movement is the roll angle. The increase of acceleration along the Y-axis somehow compensated for a reduction in the X acceleration, probably due to the greater efforts of the accessory muscles that also reduced the roll and pitch movements. Therefore, the SENSIRIB data acquisition detected a shift toward accessory muscle-centered respiration following surgery. This typical respiratory pattern could be explained by diaphragmatic palsy. Nonetheless, pain alters the respiratory pattern, leading to shorter and more superficial breaths, leading to respiratory fatigue and accessory muscle use. As aforementioned, a heterogeneity could be found in the inter-respiratory act analysis in the post-operative period. This heterogeneity could be due to the antalgic mechanism. As the use of the accessory muscles requires a greater muscular effort, the patient alternates the diaphragmatic and accessory muscle-based breaths. The former diaphragmatic breaths are deeper and allow better oxygenation as well as respiratory fatigue relief but are painful due to the compression of the lung among the chest tube, which elicits parietal pleural nociceptive neural fibers. The latter are superficial and characterized by slow and controlled inspiratory phases not to elicit pain followed by a rapid relaxation of the muscles, explaining the rapid expiratory phase. These controlled and rigid respiratory acts require a great muscular energy expense. Therefore, the patient tries to reduce inspiratory pain by gradually increasing energy consumption until respiratory fatigue forces the patient to take a deeper diaphragmatic breath. All these effects were recognized in the data that was acquired by the SENSIRIB device, as in the examples in Figure 6 and Figure 7.

### 4.2. Post-Operative Complications

The analysis of the SENSIRIB data in patients undergoing post-operative atelectasis showed several noticeable aspects. Firstly, a comparison could be made between the pre-operative and post-operative data measured on the operated hemithorax. Under this perspective, a quantitative analysis showed a decrease in both the roll and pitch angles and in the acceleration values. This reduction in all the measured parameters characterized the decrease in the chest wall motion that happens in atelectasis or other respiratory failure causes. A qualitative analysis showed post-operative curves with identified inspiratory and expiratory phases. The disarrangement in the post-operative condition as indicated in the plot curves demonstrated an ineffective chest wall expansion of the operated hemithorax. When the post-operative data collected on the non-operated hemithorax were compared with the pre-operative values, the acceleration along the X-axis and the pitch angle were found to have similar values. The conservation of the pre-operative respiratory pattern could demonstrate an attempt of the non-operated hemithorax to compensate for the atelectatic-operated lung. To maintain adequate oxygenation, both the diaphragm and the accessory muscles are used during respiration despite surgery-related pain. Nonetheless, the roll angle range was larger in the non-operated hemithorax when compared with the operated one but it was decreased when compared with the pre-operative condition. Conversely, no relevant differences were found in the pitch angle of the non-operated hemithorax with respect to the pre-operative condition. As described in other works, this result demonstrated a reduction in the diaphragmatic contraction due to surgically induced diaphragmatic palsy in the non-operated hemithorax. A reduction in the hemithorax motion is the first alert of atelectasis that, if neglected, can lead to pneumonia, sepsis or respiratory failure, eventually requiring invasive ventilation. Therefore, the early recognition of atelectasis can lead to an increased amount of respiratory therapy, mucolytic therapy or eventually to a disobstructive bronchoscopy, preventing respiratory failure.

## 5. Conclusions

The reported experiences on thorax-operated patients showed that the SENSIRIB device could be successfully applied to thoracic surgery clinical armamentarium, enlightening post-operative changes in respiratory biomechanics. In addition, the SENSIRIB was reliable in the early detection of post-operative complications and in respiratory rate monitoring, as discussed in the reported illustrative test example. The main advantage of the SENSIRIB device could be summarized in a dedicated operation of respiration monitoring via the analysis of ribcage motion by using small and portable light efficient components in an integrated design and function. Future work can be planned to improve the medical protocol as a function of the additional technical features of the device for a better user-oriented operation with medical operators.

## 6. Patents

Ceccarelli, M., Portable device for measuring the movement of human ribs, patent pending n. 102021000005726, 13 March 2021, Italy.

## Figures and Tables

**Table 1:** A summary of numerical data from the test results in Figure 6 and Figure 7. The boldness indicates the parameters.

	Pre-Operation				Post-Operation			Non-Operated Site		
	Acc. X (g)	Acc. Y (g)	Acc. Z (g)		Acc. X (g)	Acc. Y (g)	Acc. Z (g)	Acc. X (g)	Acc. Y (g)	Acc. Z (g)
**Mean**	−0.85	0.54	0.07	**Mean**	−0.81	−0.33	0.05	−0.80	0.31	0.04
**SD**	0.00	0.00	0.01	**SD**	0.00	0.01	0.01	0.00	0.00	0.01
	**Roll (deg)**	**Pitch (deg)**			**Roll (deg)**	**Pitch (deg)**		**Roll (deg)**	**Pitch (deg)**	
**Mean**	2.2	0.90		**Mean**	1.50	1.10		2.10	2.3	
**SD**	2.04	0.58		**SD**	0.87	0.13		0.72	0.15	

## References

[1] Bommart S., Berthet J.P., Durand G., Ghaye B., Pujol J.L., Marty-Ané C., Kovacsik H. (2016). Normal postoperative appearances of lung cancer. Diagn. Interv. Imaging.

[2] Nonaka M., Kadokura M., Yamamoto S., Kataoka D., Iyano K., Kushihashi T., Kawada T., Takaba T. (2000). Analysis of the anatomic changes in the thoracic cage after a lung resection using magnetic resonance imaging. Surg. Today.

[3] Sengul A.T., Sahin B., Celenk C., Basoglu A. (2013). Postoperative lung volume change depending on the resected lobe. Thorac. Cardiovasc. Surg..

[4] Reilly J.J. (1995). Benefits of aggressive perioperative management in patients undergoing thoracotomy. Chest.

[5] Massard G., Wihlm J.M. (1998). Postoperative atelectasis. Chest Surg. Clin. N. Am..

[6] Peroni D.G., Boner A.L. (2000). Atelectasis: Mechanisms, diagnosis and management. Paediatr. Respir. Rev..

[7] Folke M., Cernerud L., Ekström M., Hök B. (2003). Critical review of non-invasive respiratory monitoring in medical care. Med. Biol. Eng. Comput..

[8] Krehel M., Schmid M., Rossi R.M., Boesel L.F., Bona G.L., Scherer L.J. (2014). An optical fibre-based sensor for respiratory monitoring. Sensors.

[9] Ciocchetti M., Massaroni C., Saccomandi P., Caponero M.A., Polimadei A., Formica D., Schena E. (2015). Smart Textile Based on Fiber Bragg Grating Sensors for Respiratory Monitoring: Design and Preliminary Trials. Biosensors.

[10] Ono T., Takegawa H., Ageishi T., Takashina M., Numasaki H., Matsumoto M., Teshima T. (2011). Respiratory monitoring with an acceleration sensor. Phys. Med. Biol..

[11] Yang J., Chen B., Zhou J., Lv Z. (2015). A Low-Power and Portable Biomedical Device for Respiratory Monitoring with a Stable Power Source. Sensors.

[12] Beyer B., Van Sint Jan S., Chèze L., Sholukha V., Feipel V. (2016). Relationship between costovertebral joint kinematics and lung volume in supine humans. Respir. Physiol. Neurobiol..

[13] Kondo T., Kobayashi I., Taguchi Y., Hayama N., Tajiri S., Yanagimachi N. (2005). An analysis of the chest wall motions using the dynamic MRI in healthy elder subjects. Tokai J. Exp. Clin. Med..

[14] Ceccarelli M. (2021). Portable Device for Measuring the Movement of Human Ribs. Italy Patent.

[15] Ceccarelli M., Puglisi L., Mesiti F., Ambrogi V. A device for experimental characterization of biomechanics of breathing and coughing. Proceedings of the 26th ABCM International Congress of Mechanical Engineering.

[16] Cretikos M.A., Bellomo R., Hillman K., Chen J., Finfer S., Flabouris A. (2008). Respiratory rate: The neglected vital sign. Med. J. Aust..

[17] Churpek M.M., Yuen T.C., Park S.Y., Meltzer D.O., Hall J.B., Edelson D.P. (2012). Derivation of a cardiac arrest prediction model using ward vital signs. Crit. Care Med..

[18] Nicolò A., Massaroni C., Schena E., Sacchetti M. (2020). The Importance of Respiratory Rate Monitoring: From Healthcare to Sport and Exercise. Sensors.

